# Association of pandemic-era US county-level policies with adult health and health behaviors

**DOI:** 10.1016/j.ssmph.2026.101950

**Published:** 2026-07-11

**Authors:** Emily C. Dore, Emily Wright, Kaitlyn E. Jackson, Guangyi Wang, Matthew M. Lee, Mark J. Pletcher, Thomas W. Carton, Rita Hamad

**Affiliations:** a Department of Social and Behavioral Sciences, Harvard T.H. Chan School of Public Health, Boston, MA, USA; b Institute for Health Policy Studies, University of California San Francisco, San Francisco, CA, USA; c Department of Epidemiology and Biostatistics, University of California San Francisco, San Francisco, CA, USA; d Louisiana Public Health Institute, New Orleans, LA, USA; e California Policy Lab, University of California Berkeley, Berkeley, CA, USA

## Abstract

• We examined associations of US county-level pandemic-era policies with health.
• More comprehensive containment policies were linked with better physical health.
• More comprehensive containment policies were linked with less alcohol use.
• Findings were similar for total policy comprehensiveness scores.
• These relationships varied by sex, education, race/ethnicity, and county urbanicity.

## Funding sources

Funding for this work was provided by the Patient-Centered Outcomes Research Institute (grant number COVID-2020C2-10761) and the National Institute of Mental Health (grant number U01MH129968). Dr. Lee was supported by a training grant (5T32HL098048) from the National Heart, Lung, and Blood Institute. Dr. Dore was supported by a Loan Repayment Program award from the National Institutes of Child Health and Human Development (L60HD119763). The funders had no role in the design of the study; collection, analysis, and interpretation of data; or writing the manuscript.

## Declaration of interest

The authors have no competing interests or financial disclosures.

## 1 Introduction

Health varies by county in the United States (US), leading to disparities in life expectancy as wide as 25 years between counties with the lowest and highest life expectancies (County Health Rankings & Roadmaps, 2024; Sylte et al., 2025). Local policies contribute to place-based disparities by providing resources and opportunities for good health that differ based on jurisdiction (Montez et al., 2021). For example, state social policies, including states’ earned income tax credit and paid family leave programs, are associated with improved health, including decreased alcohol use and better mental health (Bullinger, 2019; Doran et al., 2020; Morgan et al., 2020, 2022; Shields-Zeeman et al., 2021). Local policy variation, including at the county level, during the COVID-19 pandemic may also have been impactful given the societal upheaval caused by the pandemic and the tremendous variation in policymaking during this period (Chiang et al., 2025; Hamad et al., 2022).

Policies intended to prevent COVID-19 morbidity and mortality and to mitigate the pandemic's economic fallout may also have affected other health outcomes beyond COVID-19. First, containment and closure policies meant to reduce COVID-19 infections were hypothesized to increase social disconnectedness, which in turn can be harmful to psychosocial health and health behaviors (Klinenberg & Leigh, 2024; Steptoe et al., 2013). Studies have found that state-wide social distancing policies were associated with increased drug overdose mortality, worsened mental health, and reduced health care access (Chiesa et al., 2021; Devaraj & Patel, 2021; Monnat et al., 2023; Wolf et al., 2024; Yao et al., 2022). One study found that state-level physical distancing laws were associated with reduced suicide rates for men but not for women (Wiemers et al., 2025).

Like containment and closure policies, local economic policies implemented to mitigate pandemic-era economic hardship may have also had wider-ranging impacts on other health measures in addition to alleviating financial insecurity. For example, economic supports are associated with decreased stress, depression, and anxiety (Donnelly & Farina, 2025), conditions often tied to material hardship. However, extra income is also hypothesized to facilitate access to alcohol and cigarettes, which are sometimes used as copying mechanisms during times of stress (Friedman, 2020). National and state-level economic policies implemented during the COVID-19 pandemic—including the 2021 expanded Child Tax Credit, easing of rules for Temporary Assistance for Needy Families, eviction moratoria, stimulus checks, and paid sick leave—were associated with decreased smoking and binge drinking, improved mental health, and increased likelihood of good general and physical health during the pandemic (Ali & Wehby, 2022; Batra et al., 2023; Chu & Teng, 2022; Donahoe et al., 2025; Donnelly et al., 2024; Dore et al., 2022; Pignatti & Parolin, 2024; Rook et al., 2023; Schnake-Mahl et al., 2022; Yao et al., 2022).

Lastly, policies related to promoting public health may have also indirectly affected health outcomes beyond COVID-19 infection. Public health policies aim to prevent the spread of illness, and therefore could have potentially decreased individuals’ fears of becoming ill from COVID-19. These policies include educational campaigns about COVID-19, contact tracing, vaccine availabilities, and mask mandates. For example, there is some evidence of an association between public health policies, including state-level vaccine policies and national mask mandates, with improved mental health (Agrawal et al., 2021; Hallyburton et al., 2024). While studies of these policies have indicated their utility in improving health, less is known about how local policies might have exacerbated or buffered the negative impacts of the COVID-19 pandemic.

Policies also often have different effects based on sociodemographic and contextual characteristics, due to structural factors that result in differences in take-up, access, and utilization (Cintron et al., 2023). Pandemic experiences differed based on these characteristics, making exposure to these policies more or less salient. For example, greater caregiving demands during the pandemic disproportionately harmed women (Brown et al., 2022; Goldin, 2022); as a result, containment and closure policies (e.g., school closures) and public health policies (e.g., vaccine availability) may have been differentially experienced by women compared to men. Other differences may be due to different responses to the policies. For example, Republicans are more likely to value individual freedom and less government involvement than Democrats (Silver & van Kessel, 2021), which may have contributed to more resistance to mask mandates among conservatives and in more Republican areas (Dillard et al., 2021; Rains et al., 2022). In addition, men and people in rural areas were less likely to wear masks, and Black and Hispanic individuals were less likely to be vaccinated for COVID-19 (Haischer et al., 2020; Latkin et al., 2021).

To date, studies on the health effects of policies implemented during the COVID-19 pandemic within the US have mainly focused on national or state-level policies. The few studies at the local level have been limited to singular policy exposures, smaller geographies (e.g., singular states or cities), or shorter time periods over the course of the pandemic (Hurt et al., 2023; Ritchie et al., 2021; Wright et al., 2025). Few studies, to our knowledge, have leveraged local-level policy data across multiple pandemic-era policy domains to assess the association of pandemic-era policies with health in the US. The current study expands on this research to examine the 1) association of county-level COVID-19 policy comprehensiveness with health and health behaviors, and 2) whether associations varied by individual-level sociodemographic and county-level contextual characteristics. It thereby informs local policies during future public health crises.

## 2 Methods

### 2.1 Study sample

This study involved linking individual-level health data with county-level policy data. Individual health data were drawn from the Behavioral Risk Factor Surveillance System (BRFSS) for participants interviewed in 2020-2021 (N = 1,283,919). BRFSS is a serial cross-sectional nationally representative telephone survey administered each year to US non-institutionalized adults. It collects information on health behaviors, health outcomes, and demographic characteristics. Unlike other national surveys that halted data collection and/or introduced new survey modalities during the COVID-19 pandemic, BRFSS continued phone-based data collection and reported only slight reductions in response rates (47.9% in 2020 and 44.0% in 2021 compared to 49.4% in 2019) (Centers for Disease Control and Prevention, 2020,2021a;^2022^). However, 16 states began their data collection between February and July instead of January in 2020 (Centers for Disease Control and Prevention, 2021b).

We linked restricted-use BRFSS data on respondent county of residence to the US COVID-19 County Policy (UCCP) Database (Hamad et al., 2022, 2024). The UCCP Database captures county-level policies implemented in response to the COVID-19 pandemic for each week during January 2020-December 2021. Data were abstracted by trained data collectors from online sources and included policies in three domains: containment and closure (e.g., school and workplace closings), economic support (e.g., income and housing support), and public health (e.g., facial coverings and vaccination availability). The UCCP Database includes policies enacted in 309 US counties, with at least one county in each of the 50 states and Washington, DC. The counties are not a representative sample of US counties, but over half of the US population resides in these counties, and the counties are diverse with respect to racial composition, geography, and political environment. The UCCP Database, including the county sampling strategy, a full list of the 26 policies and their measurement, is described in the Supplement and in a freely available online data repository (Hamad et al., 2022, 2024).

The analytic sample consisted of BRFSS participants who had data on at least one health outcome of interest, had no missingness for covariates, and resided in a county included in the UCCP Database (N = 271,360) (see sample flowchart, Supplemental Fig. 1). No ethical approval was required for secondary analysis of de-identified BRFSS data or for UCCP policy data collection.

### 2.2 Measures

#### 2.2.1 Exposure

We used data from the UCCP Database to construct the policy exposures. Each of the 26 individual policies was scored based on its comprehensiveness, with values of 0 representing no restrictions (or no policy), 1 representing the most comprehensive policy, and values between 0 and 1 representing policies of intermediate comprehensiveness (see Supplemental Table 1 for a list of all policies and their scoring). In addition, for each week and county, the policy comprehensiveness scores were summed within each domain to create three domain-specific policy scores, and summed across all domains to create a total policy score. Although the three policy domains may have had opposing health effects, the total policy score represents the overall policy environment. Higher policy comprehensiveness scores indicated greater comprehensiveness (i.e., more stringent containment/closure requirements; more generous economic support; more extensive public health measures). The range of possible values depended on the number of possible policies in place by domain: 0-13 for containment/closure policies, 0-5 for economic response policies, and 0-8 for public health policies.

Our final exposure variables were the average of these county and week specific policy scores in the 8 weeks prior to the interview for each person. Most outcome variables reflected health and health behaviors 30 days before the interview (described below). Thus the 8-week policy exposure score captured the policy context during this 30-day window and the month prior.

#### 2.2.2 Outcomes

Outcomes included measures of health and health behaviors that are likely to be responsive to policies in the short-term, including alcohol and smoking behaviors, exercise, and self-reported measures of physical and mental health. Specifically, we examined the following self-reported continuous measures of health within the past 30 days: number of poor physical health days and number of poor mental health days. We also examined dichotomous measures of health and behaviors within the past 30 days: any exercise, current smoking, any alcohol use, any binge alcohol use (≥4 drinks on one occasion for women, ≥5 for men), any heavy alcohol use (>7 drinks per week for women, >14 for men), and frequent mental distress (≥14 days of poor mental health). In general, these measures are either defined by BRFSS and are similar to the National Institute on Alcohol Abuse and Alcoholism's definitions (e.g., heavy and binge alcohol use) and/or were based on validated measures (e.g., frequent mental distress) (Liu et al., 2018; National Institute on Alcohol Abuse & Alcoholism, 2025). See Supplemental Methods for more details on the outcome measures.

#### 2.2.3 Covariates

Covariates included categorical variables for age, sex, race/ethnicity, educational attainment, employment status, insurance status, and marital status. We used the imputed race/ethnicity variable from BRFSS, with values derived either from a participant-reported race/ethnicity, or when participants refuse to respond (<3%), the most common race/ethnicity response for that region of the state. While this imputation approach may have resulted in some misclassification, we used this variable to retain as many observations as possible. Categories included Non-Hispanic (NH) White, NH Black, NH Asian, Hispanic and NH Other. The latter category includes participants who reported NH American Indian/Alaskan Native, NH Native Hawaiian/other Pacific Islander, Multiracial, or “other race.” These groups are heterogeneous but were combined due to small sample sizes.

#### 2.2.4 Missingness

Missingness was 2% or less for each covariate, and 9% or less for each health outcome. Risk of bias is considered low when missingness is less than 10%, so we conducted complete case analysis (Langkamp et al., 2010).

### 2.3 Statistical analysis

#### 2.3.1 Policy score variation

First, we examined variation in policy comprehensiveness scores to ensure sufficient variation to detect a policy effect on the health outcomes, especially given our use of county and month fixed effects models that only leverage variation *within* a given county and month (described below). To do this, we first calculated the standard deviations of the policy scores. We then calculated the standard deviations of the residual values, drawn from a model that regressed the policy scores on county and month fixed effects. Then, we calculated the differences between these two sets of standard deviations, which represent the difference in variation in the policy exposure in models with and without county and month fixed effects (Mummolo & Peterson, 2018).

#### 2.3.2 Primary analysis

First, we tabulated descriptive statistics for the analytic sample of those residing in UCCP counties. We also compared these values to descriptive statistics among those residing in non-UCCP counties. Then, we regressed each outcome on the policy exposures of interest. We estimated two models: one including the three domain-specific policy comprehensiveness scores (i.e., three separate coefficients for containment/closure, economic, and public health) and the second including only the total policy comprehensiveness score. See Supplemental Methods for details on the equations.

All models controlled for the covariates listed above, as well as county and month fixed effects (i.e., indicator variables). The county fixed effects accounted for observed and unobserved time-invariant potential confounders at the county level, and the month fixed effects accounted for secular confounders (i.e., underlying temporal trends). Estimates from these models can be interpreted as the association between a 1-point increase in a given policy comprehensiveness score and either the probability of the outcome (for dichotomous outcomes) or the number of poor mental or physical health days (for continuous outcomes).

We estimated multivariable linear regression models for all outcomes. We did not estimate logistic regressions for dichotomous outcomes because of issues with convergence in the presence of many covariates (i.e., numerous county fixed effects). This approach is considered acceptable in the setting of large sample sizes likes ours and because we are not predicting outcome values (W. Greene, 2004; Timoneda, 2021). For ease of interpretation, we multiplied estimates from the linear probability models for each binary outcome by 100 and report them as percentage-point changes in probability. We clustered standard errors at the state level to account for correlated observations across counties within the same state. We did not apply survey weights to the analysis because the UCCP Database is not a nationally representative sample of US counties and, thus, we are not presenting population-level estimates.

#### 2.3.3 Subgroup analyses

We also assessed heterogeneous associations between the policy scores and health outcomes by subgroups. These analyses are important given evidence of the heterogeneous effects of policies by subgroup due to different pandemic experiences and responses to pandemic policies, as described above. At the individual level, we assessed associations by sex, educational attainment, and race/ethnicity. At the county level, we assessed associations by urbanicity (defined as “hyper urban” if the percentage of the county population living in urban areas was ≥95%) and political partisanship (operationalized as the share of Democratic votes in the 2016 presidential election, split at the median, 47.3%). For these analyses, we estimated models stratified by each aforementioned group.

#### 2.3.4 Sensitivity analyses

We conducted two sensitivity analyses. The first included county-level COVID-19 mortality rate as a covariate in the main model to rule out disease severity as a confounder of the relationship between the policies and health outcomes. The second examined the association between a selection of individual policy scores hypothesized to be especially meaningful (e.g., stay-at-home orders and income support policies) and health outcomes. Results are in the Supplement.

## 3 Results

### 3.1 Sample characteristics

Among the 271,360 individuals in the UCCP study sample, 30.6% were 65 years or older (Table 1). Most were White (65.8%), and nearly half had graduated from college or technical school (46.9%). A majority were employed (54.6%) and married or partnered (53.7%). Almost all respondents had health insurance at the time of the survey (92.5%). See Supplemental Table 2 for details on BRFSS participants who resided in non-UCCP counties.

**Table 1:** Sample characteristics, 2020-2021.

	Mean (SD) or Percent
N	271,360

*Covariates*	

Age (years)	
18-24	7.1
25-34	13.3
35-44	14.8
45-5	16.2
55-64	17.9
65+	30.6
Women	53.4
Race/ethnicity	
NH White	65.8
Hispanic	12.7
NH Black or African American	11.9
NH Asian	4.6
NH Other^a^	5.1
Education	
High school or less	27.6
Some college or technical school	25.6
Graduated college or technical school	46.9
Employed	54.6
Health insurance coverage	92.5
Married or in a couple	53.7

*Outcomes*^b^	

Frequent mental distress	12.9
Any exercise	78.8
Current smoker	11.3
Any alcohol use	54.7
Any binge alcohol use	14.0
Any heavy alcohol use	6.2
Number of poor physical health days	3.3 (7.8)
Number of poor mental health days	4.2 (8.1)

Abbreviations: BRFSS = Behavior Risk Factor Surveillance System; UCCP = US COVID-19 County Policy Database; NH = Non-Hispanic, SD = standard deviation. Table presents percentages for categorical variables and mean (standard deviation) for continuous variables.

a NH Other includes the following groups collapsed due to small sample size: American Indian or Alaskan Native, Native Hawaiian or other Pacific Islander, Multiracial, and “other race” groups.

b All outcomes are in reference to the past 30 days, except current smoker which asks about smoking behaviors practiced “now.”

### 3.2 Variation in policy comprehensiveness scores

Standard deviations for the original policy scores ranged from 1.4 for economic policy comprehensiveness to 3.1 for containment/closure policy comprehensiveness (Supplemental Table 3). Standard deviations for the residuals, which accounted for county and month fixed effects, were between 0.5 for public health policy comprehensiveness and 1.2 for containment/closure policy comprehensives. Thus, as expected, there was some loss in variation in the policy exposure in models that accounted for fixed effects. Nonetheless, there remained sufficient variation, and the inclusion of county and month fixed effects controls for unobserved place and time characteristics and facilitates better causal conclusions.

### 3.3 Primary analyses

**Fig. 1:**
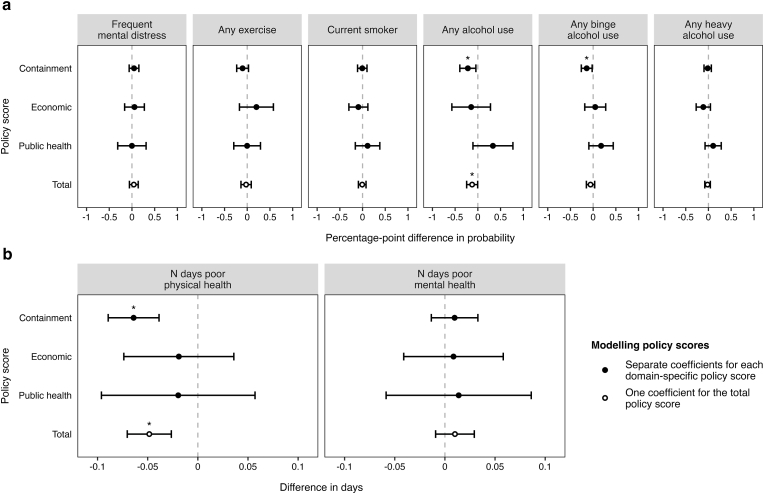
Association of Policy Comprehensiveness Scores with Health, BRFSS-UCCP data 2020-2021 (n = 271,360). Note: ∗p < 0.05. BRFSS = Behavioral Risk Factor Surveillance System; UCCP = US COVID-19 County Policy Database. Results are from fully adjusted county and month fixed effects models regressing each a) binary health outcome and b) continuous health outcome on the three domain-specific policy scores and, separately, total policy score. All models were adjusted for age, sex, race/ethnicity, education, employment status, health insurance status, and marital status. Whiskers indicate 95% confidence intervals.

In our primary analysis (Fig. 1), a 1-point increase in the containment/closure policy comprehensiveness score was associated with a lower likelihood of any alcohol use (−0.22 percentage points [pp], 95%CI -0.40, −0.04) and binge alcohol use (−0.14pp, 95%CI -0.26, −0.02), as well as fewer poor physical health days (−0.06 days, 95%CI -0.09, −0.04). Economic and public health policy comprehensiveness scores were not associated with health outcomes in the overall sample. A 1-point increase in the total policy comprehensiveness score was associated with lower likelihood of any alcohol use (−0.13pp, 95%CI -0.25, −0.004) and fewer poor physical health days (−0.05 days, 95%CI -0.07, −0.03).

### 3.4 Subgroup analyses

In stratified subgroup analyses, there were some estimates for an individual group (e.g., women) that were different from zero, but the estimates were rarely different from the other groups (e.g., point estimates for women's estimates were included in confidence intervals for men's estimates). There were no consistent patterns across subgroups, but we discuss some examples in the following paragraphs.

**Fig. 2:**
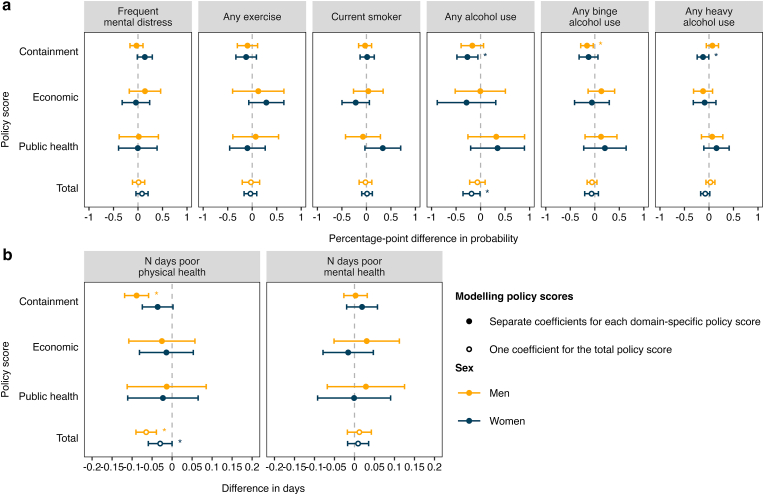
Association of Policy Comprehensiveness Scores with Health, by Sex, BRFSS-UCCP data 2020-2021 (n = 271,360). Note: ∗p < 0.05. BRFSS = Behavioral Risk Factor Surveillance System; UCCP = US COVID-19 County Policy Database. Results are from fully adjusted county and month fixed effects models regressing each a) binary health outcome and b) continuous health outcome on the three domain-specific policy scores and, separately, total policy score, by sex. All models were adjusted for age, race/ethnicity, education, employment status, health insurance status, and marital status. Whiskers indicate 95% confidence intervals.

In models stratified by sex, containment/closure and total policy comprehensiveness scores had differing associations with alcohol use and physical health among men and women participants (Fig. 2). More specifically, more comprehensive containment/closure policy scores were associated with lower likelihood of any alcohol use and heavy alcohol use for women, and any binge alcohol use for men. More comprehensive total policy scores were associated with a decreased number of poor physical health days for both men and women, but men had a larger decrease.

**Fig. 3:**
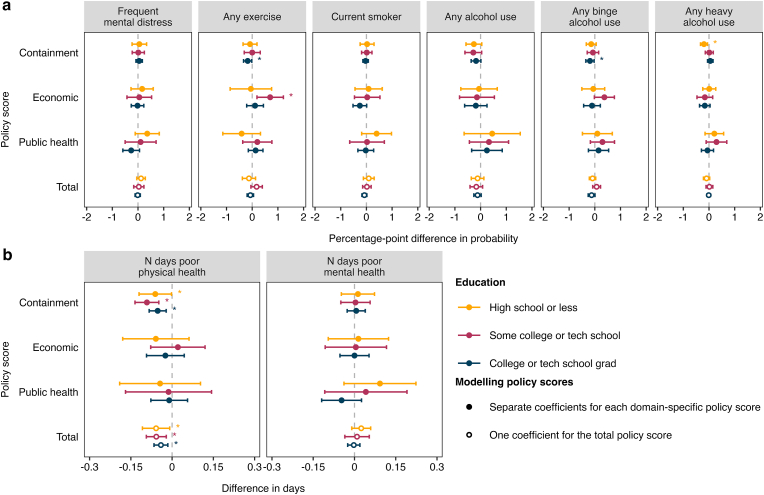
Association of Policy Comprehensiveness Scores with Health, by Education, BRFSS-UCCP data 2020-2021 (n = 271,360). Note: ∗p < 0.05. BRFSS = Behavioral Risk Factor Surveillance System; UCCP = US COVID-19 County Policy Database. Results are from fully adjusted county and month fixed effects models regressing each a) binary health outcome and b) continuous health outcome on the three domain-specific policy scores and, separately, total policy score, by education. All models were adjusted for age, sex, race/ethnicity, employment status, health insurance status, and marital status. Whiskers indicate 95% confidence intervals.

In models stratified by education, more comprehensive containment/closure and total policy scores were associated with fewer poor physical health days for all groups (Fig. 3). Otherwise, there were isolated differential associations between policy scores and alcohol use across educational groups. For example, more comprehensive containment/closure policy scores were associated with lower likelihood of heavy alcohol use among individuals with a high school education or less, and more comprehensive education policy scores were associated with higher likelihood of exercise for individuals with some college or technical school.

**Fig. 4:**
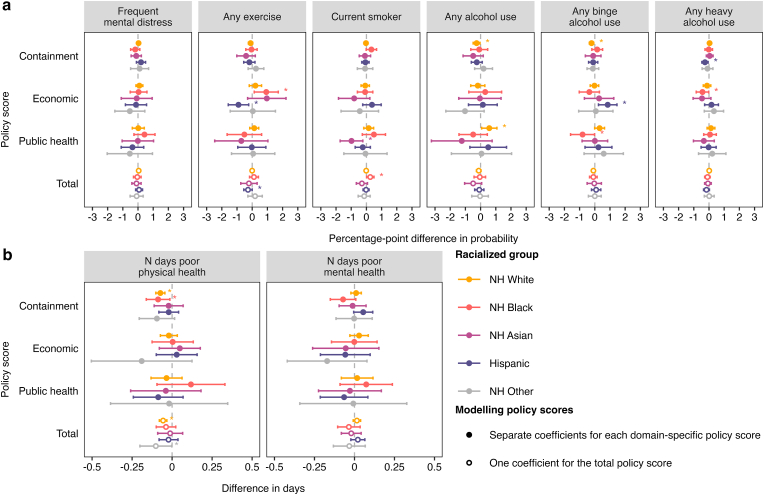
Association of Policy Comprehensiveness Scores with Health, by Race/Ethnicity, BRFSS-UCCP data 2020-2021 (n = 271,360). Note: ∗p < 0.05. BRFSS = Behavioral Risk Factor Surveillance System; UCCP = US COVID-19 County Policy Database. Results are from fully adjusted county and month fixed effects models regressing each a) binary health outcome and b) continuous health outcome on the three domain-specific policy scores and, separately, total policy score, by race/ethnicity. All models were adjusted for age, sex, education, employment status, health insurance status, and marital status. Whiskers indicate 95% confidence intervals.

In models stratified by race/ethnicity, more comprehensive containment/closure and total policy scores were associated with less alcohol use and fewer poor physical health days for many groups; however, economic and public health policy comprehensiveness had mixed associations across groups (Fig. 4). More specifically, more comprehensive containment/closure policy scores were associated with lower likelihood of any alcohol use and binge alcohol use for White participants, and lower likelihood of heavy alcohol use for Hispanic participants. More comprehensive containment/closure scores were associated with fewer poor physical health days for White and Black participants, while more comprehensive total policy scores were associated with fewer poor physical health days for White and Other race participants. More comprehensive economic policy scores were associated with worse health outcomes for Hispanic adults, including less exercise and higher likelihood of binge alcohol use. More comprehensive public health scores were also associated with decreased binge alcohol use among Black participants.

**Fig. 5:**
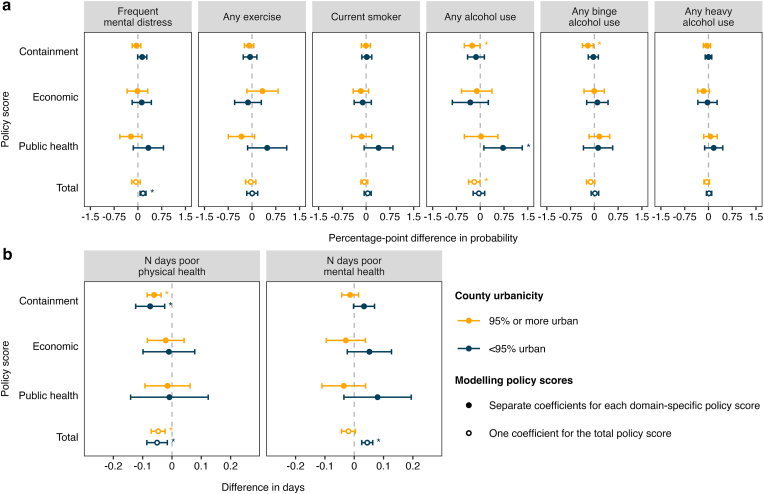
Association of Policy Comprehensiveness Scores with Health, by County Urbanicity, BRFSS-UCCP data 2020-2021 (n = 271,360). Note: ∗p < 0.05. BRFSS = Behavioral Risk Factor Surveillance System; UCCP = US COVID-19 County Policy Database. Results are from fully adjusted county and month fixed effects models regressing each a) binary health outcome and b) continuous health outcome on the three domain-specific policy scores and, separately, total policy score, by urbanicity. All models were adjusted for age, sex, race/ethnicity, education, employment status, health insurance status, and marital status. Whiskers indicate 95% confidence intervals.

In models stratified by county urbanicity, results followed a similar pattern for alcohol use and poor physical health days, but different patterns for poor mental health days (Fig. 5). For example, more comprehensive containment/closure and total policy scores were associated with fewer poor physical health days for both types of counties. More comprehensive containment/closure and total policy scores were also associated with decreased likelihood of any alcohol use for more urban counties. However, more comprehensive total policy scores were associated with higher likelihood of frequent mental distress and more poor mental health days only for less urban counties.

**Fig. 6:**
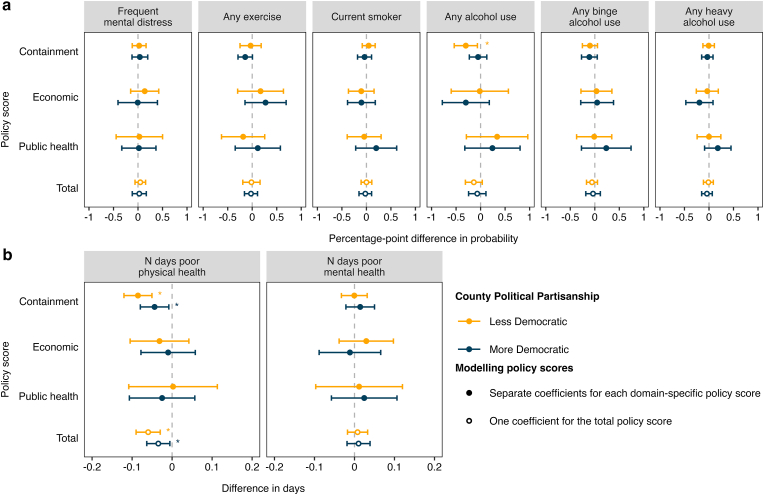
Association of Policy Comprehensiveness Scores with Health, by County Political Partisanship, BRFSS-UCCP data 2020-2021 (n = 271,360). Note: ∗p < 0.05. BRFSS = Behavioral Risk Factor Surveillance System; UCCP = US COVID-19 County Policy Database. Results are from fully adjusted county and month fixed effects models regressing each a) binary health outcome and b) continuous health outcome on the three domain-specific policy scores and, separately, total policy score, by county political partisanship. All models were adjusted for age, sex, race/ethnicity, education, employment status, health insurance status, and marital status. Whiskers indicate 95% confidence intervals.

In models stratified by county political partisanship (Fig. 6), results show very few associations between policy scores and the outcomes. More comprehensive containment/closure policy scores were associated with lower likelihood of alcohol use in less Democratic counties. More comprehensive containment/closure and total policy scores were associated with fewer poor physical health days in both types of counties, although less Democratic countries showed the larger decrease.

## 4 Discussion

This study examined the association of US pandemic-era county-level policies with health and health behaviors by linking a comprehensive set of county-level policy exposures with individual-level health outcomes in a large national sample. Overall, we found few associations between the pandemic-era policies and health outcomes, with the exception that more comprehensive containment/closure and total policy scores were associated with decreased alcohol use and fewer poor physical health days. We found no evidence of associations between economic response and public health policies and health for the overall sample. In analyses stratified by sociodemographic and county-level characteristics, associations between policy comprehensiveness scores and health varied across groups in both strength and direction. For example, more comprehensive containment/closure policy scores were associated with *decreased* heavy alcohol use for women but not men. In addition, more comprehensive containment/closure policy scores were associated with worse mental health for less urban counties, but not more urban counties. However, in general, there were few consistent patterns that one type of policy was more beneficial or more harmful for a given group or outcome.

There are a few possible explanations for why we found that more comprehensive containment/closure and total policies were associated with decreased alcohol use and fewer poor physical health days. Stay-at-home orders increased time with loved ones, which can be beneficial for health (Umberson et al., 2018; Umberson & Montez, 2010). Containment/closure policies also likely limited access to alcohol through the closing of bars and restaurants (Callinan et al., 2021; Castaldelli-Maia et al., 2021). They may also have improved physical health by limiting exposure to pathogens, including (but not limited to) COVID-19. County-level containment/closure policies seemed to be particularly effective at reducing mobility, even in the presence of state-level policies (Goolsbee & Syverson, 2021; Gupta et al., 2021).

The literature on the effects of COVID-19-related policies on health and health behaviors (beyond COVID-19 infection itself) is scarce, and the evidence available is mixed. One other study that linked the UCCP Database to health outcomes found no association between policy comprehensiveness and behavioral health, including similar alcohol use and mental health outcomes as those in the current study (Wright et al., 2025). However, that study only examined 2021 when most of the variation in policy scores represented their de-implementation. Other studies have found associations between state-level COVID-19 containment policies and increased drug-related mortality and worse mental health (Devaraj & Patel, 2021; Monnat et al., 2023; Wolf et al., 2024; Yao et al., 2022). However, studies have also found state-level physical distancing policies were associated with reduced suicide rates for men (Wiemers et al., 2025), while more intense COVID-19 policies were associated with reduced sharing of cannabis (Assaf et al., 2024). Further, a systematic review found that duration of stay-at-home orders during the pandemic was not associated with alcohol use (Acuff et al., 2022).

In the overall sample, we found no associations between economic policy comprehensiveness and health. While research on the impacts of pandemic-era county-level economic policies on health is limited, research on federal pandemic-era economic policies has generally found positive associations between these policies and health, including the 2021 Child Tax Credit expansion, changes to the Temporary Assistance for Needy Families program, and unemployment insurance (Batra et al., 2023; Berkowitz & Basu, 2021; Donahoe et al., 2025; Dore et al., 2022; Gennetian & Gassman-Pines, 2024; Jeong & Fox, 2023; Rook et al., 2023). The level of support at the county level was smaller compared to support at the state or national level, which may explain our null results for this policy domain. For example, there was a 15% increase in the Supplemental Nutrition Assistance Program at the national level, but counties’ nutrition assistance programs largely consisted of sporadic free meals, were often only for children, and required traveling to a specific location within a specific timeframe (Food and Nutrition Service, 2023; Jackson et al., 2024; Wells et al., 2024). Similarly, while federal eviction moratoria prevented evictions, many of the county-level housing support policies were more limited in their range (e.g., capped the amount of rental or utility support they offered or only offered support for a more limited period) (Greene & Batko, 2020). Even when policies were more equivalent, e.g., eviction moratoria, the county policy had a smaller effect than the state policy (An et al., 2021).

Alternatively, the group affected may have been too small to show population-level improvements since economic policies often targeted particular populations based on income (Greene & Batko, 2020). In addition, in some cases, subgroup analyses showed associations in opposite directions that cancelled each other out. For example, the association between the economic support policy comprehensiveness score and exercise were in the opposite direction for Black and Hispanic individuals.

For public health policies, studies have found that national mask mandates and state-level policies announcing vaccine eligibility were associated with improved mental health, likely due to decreased fears of infection, hospitalization, and death (Agrawal et al., 2021; Hallyburton et al., 2024). In our general sample, we found no association between public health policy comprehensiveness and any health outcomes. It is possible that county-level policies were less impactful than state or federal level policies that, for example, determined vaccine eligibility at the beginning of the vaccine rollout, or perhaps less impactful than school or workplace place policies later in the pandemic. In general, states can engage in preemption, which limits county-level governing and affects public health policies including tobacco control and firearm safety (Pomeranz & Pertschuk, 2017). This happened during the pandemic when governors prohibited local governments from enacting policies related to masking and vaccine mandates, which likely minimized the variation across counties within the same state (Markowitz & Rough, 2023).

We also found important subgroup differences, which may be due to how individuals experienced the pandemic or responded differently to policy impacts. For example, more comprehensive containment/closure policy scores were associated with decreased heavy alcohol use among women but not men. This may be related to women's greater caregiving burden when schools and daycares were closed, as women may have been too busy or exhausted to consume alcohol, or gendered differences in the relationship between stress (which could be affected by these policies) and substance use (Goldin, 2022; Verplaetse et al., 2018).

Another subgroup difference was based on county urbanicity: more comprehensive total policy scores were associated with more poor mental health days in less urban counties, but not in more urban counties. While rural counties experienced worse mental health during the pandemic overall (Monnat, 2021), mental illness may have been exacerbated due to containment and closure measures that increased isolation in already remote areas (Steptoe et al., 2013; Vogelsang, 2016). Public health policies in more urban counties may have been particularly beneficial in mitigating stress about contracting COVID-19 in such dense areas, especially since urban residents were more likely to practice preventive behaviors like wearing masks (Callaghan et al., 2021; Haischer et al., 2020).

This study contributes to the understanding of the health effects of local COVID-19-related policies using a national data set and novel policy database, although there are some limitations. First, we were limited in our ability to assess a causal relationship between individual policies and health, because there were a large number of policies enacted concurrently within a short period of time. While studying the effects of a single policy is important, co-occurring policies make isolating single policy effects difficult when they are implemented at the same time, as they were during the pandemic (Matthay et al., 2021). Nevertheless, these composite measures captured the overall policy context as opposed to a specific policy, which is itself an important research question (Montez & Grumbach, 2023). Relatedly, legislatures are increasingly becoming more polarized and tend to pass laws in bundles that result in more conservative or progressive policy contexts, which are important for health (Krieger et al., 2024; Montez et al., 2022; Wiemers et al., 2025). At the same time, our inclusion of county-level fixed effects enables within-county comparisons to avoid comparing counties with different baseline characteristics to one another. Relatedly, state-level policies that changed during the study period may have confounded the relationship between the county-level policies and health, which would help to explain the null findings for economic support and public health policy domains. Finally, the UCCP Database is not nationally representative, and results may not be generalizable to the entire US population. However, the UCCP Database includes policy information for over half of US counties that are diverse in terms of racial composition, political partisanship, and geography.

In summary, this study found that more comprehensive containment/closure and total policy scores were associated with less alcohol use and fewer poor physical health days. While we found that there was no association between economic support and public health policies and any of the health outcomes in the full sample, the many higher-level economic and public health policies implemented during the study period may have superseded the effects of the county-level policies, suggesting these results are specific to the COVID-19-era. Nonetheless, there was some variation in results across individual sociodemographic subgroups and county characteristics across all policy domains. Our findings add to the literature on COVID-19-era policies and provide evidence for potential downstream health consequences. More research is needed to understand the effects of specific policies, including for other health outcomes, long-term implications, and the mechanisms connecting these policies to health.

## Ethical statement

Our analysis of secondary, de-identified BRFSS data was deemed not human subjects research and thus no IRB application was required.

## CRediT authorship contribution statement

**Emily C. Dore:** Formal analysis, Writing – original draft. **Emily Wright:** Formal analysis, Visualization, Writing – review & editing. **Kaitlyn E. Jackson:** Formal analysis, Writing – review & editing. **Guangyi Wang:** Formal analysis, Writing – review & editing. **Matthew M. Lee:** Formal analysis, Writing – review & editing. **Mark J. Pletcher:** Conceptualization, Funding acquisition, Methodology, Writing – review & editing. **Thomas W. Carton:** Conceptualization, Funding acquisition, Methodology, Writing – review & editing. **Rita Hamad:** Conceptualization, Funding acquisition, Methodology, Supervision, Writing – review & editing.

## Conflict of interest

The authors declare no conflict of interest.

## Data Availability

The UCCP Database used for this study is publicly and freely available to any investigator via the Inter-university Consortium for Political and Social Research (ICPSR): https://doi.org/10.3886/ICPSR39109.v1. The BRFSS data used for this study are maintained as restricted-access data by the National Center for Health Statistics (NCHS) Research Data Center (RDC) and are, thus, only available to investigators who request and receive approval from NCHS to access restricted-use data files, as described here: https://www.cdc.gov/rdc/index.html.
