# Supplementary material for: Association of pandemic-era US county-level policies with adult health and health behaviors
Source: SSM Popul Health. 2026 Jul 11;35:101950. doi: 10.1016/j.ssmph.2026.101950 (PMC13382331; doi:10.1016/j.ssmph.2026.101950)
Supplement: Multimedia component 1 [file mmc1.docx]

**Supplemental Methods**

*Outcome Measures*

In the Behavioral Risk Factor Surveillance System (BRFSS) (CDC, 2024a), the number of poor physical health days was asked: “Now thinking about your physical health, which includes physical illness and injury, for how many days during the past 30 days was your physical health not good?” For the number of poor mental health days, participants were asked: “Now thinking about your mental health, which includes stress, depression, and problems with emotions, for how many days during the past 30 days was your mental health not good?” Respondents responded to both questions on a scale of 1 to 30 days. We coded the frequent mental distress variable based on the number of poor mental health days reported by dichotomizing the variable at a validated cut point of 14 days (i.e., < 14 days was not frequent mental distress, and ≥ 14 days was frequent mental distress) (Liu et al., 2018).

For any exercise in the past month, BRFSS asked “during the past month, other than your regular job, did you participate in any physical activities or exercises

such as running, calisthenics, golf, gardening, or walking for exercise?” Participants responded yes or no. BRFSS computed a current smoker variable based on a question that asked if the respondent now smokes every day, now smokes some days, is a former smoker, or is a never smoker. The first two categories were considered current smokers, and the latter two categories were not considered current smokers. For alcohol use, BRFSS computed variables based off questions that asked how many drinks of alcohol participants had per occasion or how many total in the past week or month. Adults were considered to have had any alcohol in the past month if they reported having at least one drink of alcohol in the past 30 days. Adults were considered to have had heavy alcohol use if men had more than 14 drinks per week and women had more than 7 drinks per week. Adults were considered to have had binge alcohol use if men had at least five drinks per occasion and women had at least four drinks per occasion. BRFSS defines these alcohol use variables this way, but the definitions are similar to those from the National Institute on Alcohol Abuse and Alcoholism (2025).

*US COVID-19 County Policy Database (UCCP)*

The goal of the UCCP Database was to systematically gather county-level policies implemented in response to the COVID-19 pandemic between 2020-2021 (Hamad et al., 2022, 2024). To determine the sample of counties, the research team first stratified counties based on the Centers for Disease Control and Prevention Social Vulnerability Index (SVI) (CDC, 2024b). The SVI bases an area’s social vulnerability on socioeconomic status, household characteristics (i.e., single-parent households), racial and ethnic minority status, and housing type and transportation (i.e., multi-unit structures and having no vehicle). The team oversampled counties above the median value of the SVI at a rate of 2:1 compared to counties below the median value. Then, within these strata, the team used a probability-proportional to size sampling method to ensure more populous counties were more likely to be included.

These steps were repeated until the sample included at least one county in each of the 50 states and Washington, DC, and at least 10% rural or non-metropolitan counties. This process resulted in 269 counties. An additional sample of 40 counties were included based on a prior PCORI-funded sample of US COVID Citizen Science Study patients (Beatty et al., 2021). The final sample in the UCCP Database was thus 309 counties. This sample was not intended to be nationally representative of all counties in the US, but to be inclusive of populations who were economically disadvantaged, Black and Latino, and in non-metropolitan areas. The counties include over half of the US population and are diverse in characteristics including racial/ethnic composition, geography, and political partisanship.

Once the counties were selected, a team of trained researchers collected information on 26 county policies related to containment and closure, economic response, and public health for each week and each county between January 2020-December 2021. Data were collected using resources such as governmental websites, news sources, and social media. For states where counties do not pass policies (e.g., public health initiatives in Massachusetts occur at the municipality level), the team captured policies enacted in the largest city within the county, as long as that city made up more than 50% of the population. When information on the county policies was not available, the research team imputed policy data based on state policies. Policies are characterized in two ways in the Database: 1) 0/1 scores for whether the policy was in place in that county that week and 2) measures of the comprehensiveness of each policy.

Comprehensiveness scores for each individual policy range from 0 to 1, with 0 being the least comprehensive, and 1 being the most. Since there were gradations in these policies, some policy scores include values between 0-1. For example, most economic support policies were scored as 0 (no policy in place) or 1 (the policy was in place). But for other policies, there were three or more levels. For example, school closures were scored as 0 (no school closures), 0.33 (recommend closing or all schools open with alterations), 0.66 (require closing only some levels or categories), and 1 (require closing all levels). See Supplemental Table 1 for all policy scores. We used these comprehensiveness scores to create the average 8-week policy exposures discussed in the manuscript. Pearson correlation coefficients for policy comprehensiveness scores were 0.09 for containment/closure and public health, 0.38 for containment/closure and economic, and 0.70 for economic and public health. For more information on the UCCP Database, please see details in the freely available online repository where the data are provided (Hamad et al., 2022, 2024).

*Analytic Approach*

We estimated two models for all health outcomes. Model 1 included all three of the domain-specific policy comprehensiveness scores (containment/closure, economic response, and public health). Model 2 included a total policy comprehensiveness score as the exposure of interest. The equations for the models are:

$$Y_{itcs}= \beta_{0}+\beta_{1}{score\_contain}_{itcs}+\beta_{2}{score\_econ}_{itcs}+\beta_{3}{score\_health}_{itcs}+\alpha_{c}+\delta_{m}+\gamma X_{itcs}+\varepsilon_{itc}s (1)$$

$$Y_{itcs}= \beta_{0}+\beta_{1}{score\_total}_{itcs}+\alpha_{c}+\delta_{m}+\gamma X_{itcs}+\varepsilon_{itcs} (2)$$

$Y_{itcs}$ represents the health outcome for individual $i$ interviewed in week $t$ in county $c$ in state $s$. $\alpha_{c}$ represents county fixed effects, $\delta_{m}$ represents month fixed effects, $X_{itcs}$ represents individual covariates, and $\varepsilon_{itcs}$ is the residual term (clustered at the state level). Analyses were conducted in Stata 18.0 (College Station, Texas) and figures were created using R version 4.4.1 (Vienna, Austria).

*Sensitivity Analyses*

We conducted two secondary analyses. First, we added a control for county-level COVID-19 mortality rates to the main model. Theoretically the COVID-19 policies may have been implemented or strengthened during surges in COVID-19. Controlling for COVID-19 severity thus helps to disentangle the association between the policies and health from the association between COVID-19 and health. These secondary results were substantively the same as the main results but somewhat attenuated (Supplemental Figure 2). For example, the relationship between total policy comprehensiveness and any alcohol use became non-significant, although the point estimates were both negative (in the main analysis: -0.22 percentage points [pp] (95%CI -0.40, -0.04) and in the secondary analysis: -0.12pp (95%CI -0.24, 0.00). This suggests that underlying disease severity may have also affected the outcomes of interest.

For the second sensitivity analysis, we assessed the relationship between a selection of single policy scores that would theoretically be especially impactful on the health outcomes of interest: stay-at-home orders, restaurant closures, bar closures, and income support policies. These policy scores, like the main exposures, were scored based on the average comprehensiveness in the 8-weeks before the respondent’s interview in a specific county. Thus, they only differed from the main exposure because they were scores for single policies, rather than an index of policies. While we found there were some associations between these policies and health outcomes, the results were generally null (Supplemental Figure 3). In one case, two containment/closure policy scores were associated with heavy alcohol use in opposite directions. More comprehensive stay at home policy scores were associated with less heavy alcohol use (-0.33 pp, 95%CI -0.62, -0.03) and more comprehensive restaurant policy scores were associated with more heavy alcohol use (0.23 pp, 95%CI 0.03, 0.44). This may have contributed to overall null findings in the main analysis, although this represents only one occurrence of conflicting directions.

Supplemental Figure 1. Sample Flowchart, Behavioral Risk Factor Surveillance System

Interviewed 2020-2021

N = 813,222

Residing in UCCP counties

N = 271,360

Non-missing covariates and at least one health outcome

N = 773,809

Survey years 2019-2021

N = 1,283,919

Note: UCCP= US COVID-19 County Policy Database. BRFSS 2019 included participants who were interviewed in the first few months of 2020, and were thus included in our sample.

Supplemental Figure 2. Association of Policy Comprehensiveness Scores with Health, BRFSS-UCCP data 2020-2021 (n = 271,360), controlling for county-level COVID-19 mortality rates
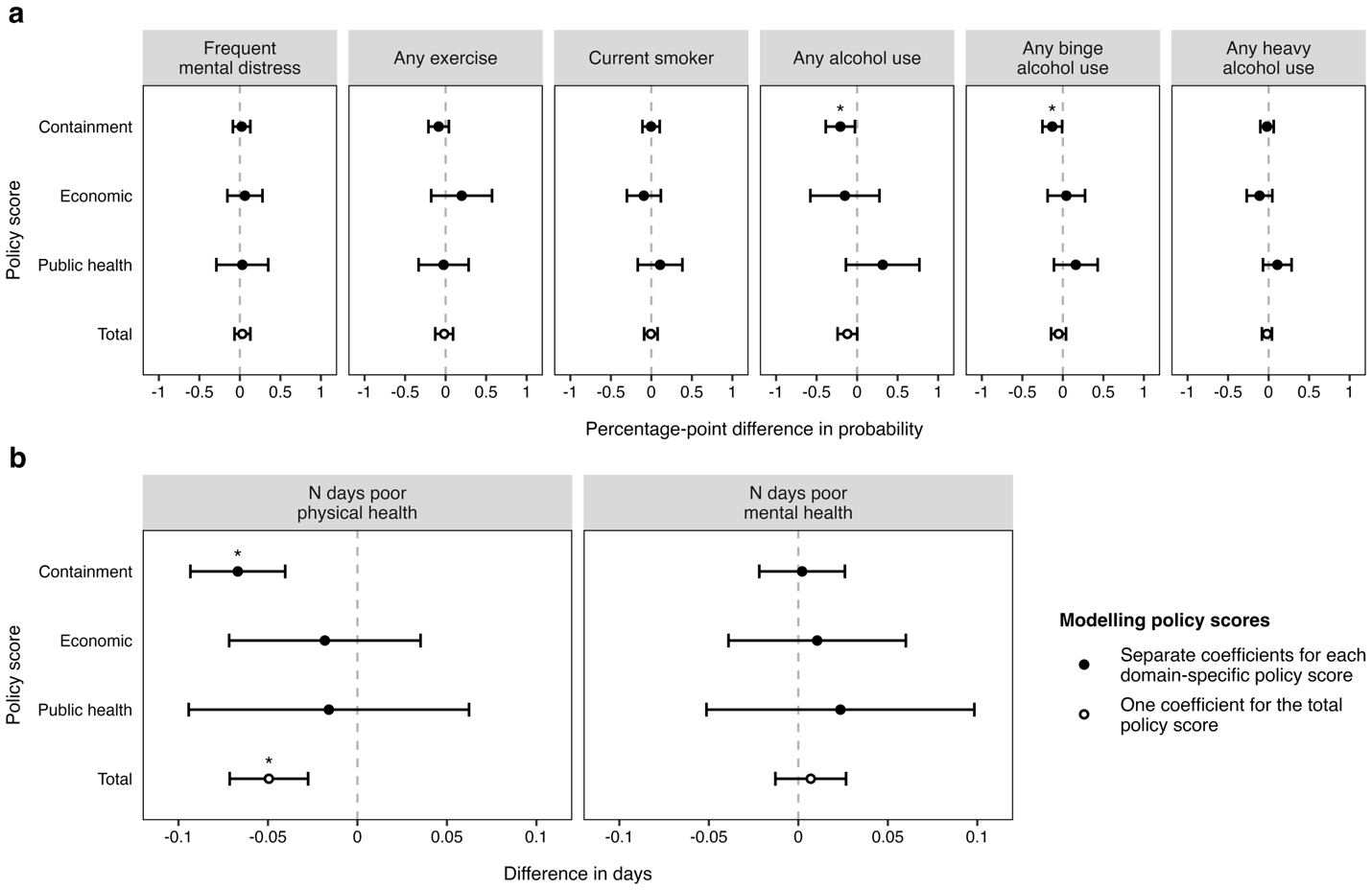


Note: * p < 0.05. BRFSS = Behavioral Risk Factor Surveillance System; UCCP = US COVID-19 County Policy Database. Results are from fully adjusted county and month fixed effects models regressing each a) binary health outcome and b) continuous health outcome on the three domain-specific policy scores and, separately, total policy score. All models were adjusted for age, sex, race/ethnicity, education, employment status, health insurance status, marital status, and county-level COVID-19 mortality rates. Whiskers indicate 95% confidence intervals.

Supplemental Figure 3. Association of Single Policy Comprehensiveness Scores with Health, BRFSS-UCCP data 2020-2021 (n = 271,360)
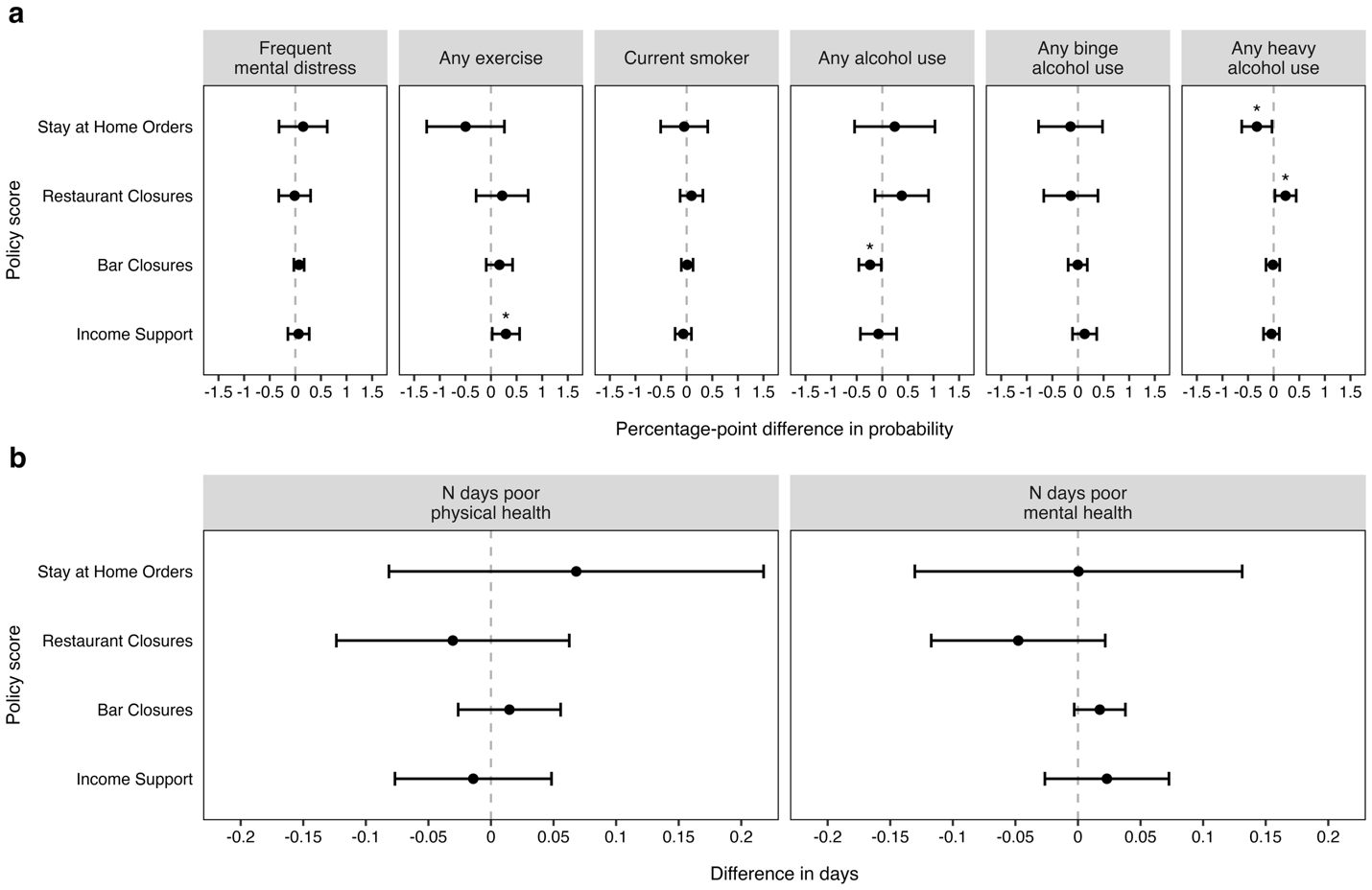
 Note: * p < 0.05. BRFSS = Behavioral Risk Factor Surveillance System; UCCP = US COVID-19 County Policy Database. Results are from fully adjusted county and month fixed effects models regressing each a) binary health outcome and b) continuous health outcome on four single policy scores. All models were adjusted for age, sex, race/ethnicity, education, employment status, health insurance status, and marital status. Whiskers indicate 95% confidence intervals.

| **Supplemental Table 1. COVID-19 county policy variables collected in the US COVID-19 County Policy Database.** | | | | |
| --- | --- | --- | --- | --- |
| **#** | **Policy** | **Original values** | **Rescaled values** | **Value labels** |
| ***Domain: Containment and closure policies*** | | | | |
| 1 | School closing | 0  1  2  3 | 0  0.33  0.66  1 | No measures (i.e., no restrictions)  Recommend closing or all schools open with alterations  Require closing only some levels or categories  Require closing all levels |
| 2 | Workplace closing | 0  1  2  3 | 0  0.33  0.66  1 | No measures (i.e., no restrictions)  Recommend closing (or recommend work from home)  Require closing (or work from home) for some non-essential workers  Require closing (or work from home) all-but-essential workplaces |
| 3 | Cancellation of public events | 0  1  2  3  4 | 0  0.25  0.50  0.75  1 | No measures (i.e., no restrictions)  Events allowed, with minimal (≥50% capacity) limitations  Events allowed, with major (<50% capacity) limitations  Recommend cancelling  Require cancelling |
| 4 | Restrictions on private gatherings | 0  1  2  3  4 | 0  0.25  0.50  0.75  1 | No measures (i.e., no restrictions)  Restrictions on very large gatherings (the limit is above 1,000 people)  Restrictions on gatherings between 101-1,000 people  Restrictions on gatherings between 11-100 people  Restrictions on gatherings of 10 people or less |
| 5 | Public transport closing | 0  1  2 | 0  0.5  1 | No measures (i.e., no restrictions)  Recommend closing (or significantly reduce volume, route, etc.)  Require closing (or prohibit most from using it) |
| 6 | Stay-at-home requirements | 0  1  2  3 | 0  0.33  0.66  1 | No measures (i.e., no restrictions)  Recommend not leaving house  Require not leaving house with exceptions for “essential” trips  Require not leaving house with minimal exceptions |
| 7 | Gym closing | 0  1  2  3 | 0  0.33  0.66  1 | No measures (i.e., no restrictions)  Open, with minimal (≥50% capacity) limitations  Open, with major (<50% capacity) limitations  Closed |
| 8 | Restaurant closing | 0  1  2  3  4  5 | 0  0.2  0.4  0.6  0.8  1 | No measures (i.e., no restrictions)  Open for indoor dining, with minimal (≥50% capacity) limitations  Open for indoor dining, with major (<50% capacity) limitations  Outdoor only (with or without takeout/delivery)  Takeout/delivery only  Closed |
| 9 | Bar closing | 0  1  2  3  4  5 | 0  0.2  0.4  0.6  0.8  1 | No measures (i.e., no restrictions)  Open for indoor drinking, with minimal (≥50% capacity) limitations  Open for indoor drinking, with major (<50% capacity) limitations  Outdoor only (with or without takeout/delivery)  Takeout/delivery only  Closed |
| 10 | Movie theater closing | 0  1  2  3 | 0  0.33  0.66  1 | No measures (i.e., no restrictions)  Open, with minimal (≥50% capacity) limitations  Open, with major (<50% capacity) limitations  Closed |
| 11 | Day care closing | 0  1  2  3 | 0  0.33  0.66  1 | No measures (i.e., no restrictions)  Recommend closing or all day cares open with alterations  Require closing (only some levels or categories - e.g., babies)  Require closing all levels |
| 12 | Restrictions on religious gatherings | 0  1  2  3 | 0  0.33  0.66  1 | No measures (i.e., no restrictions)  Open, with minimal (≥50% capacity) limitations  Open, with major (<50% capacity) limitations  Closed |
| 13 | Curfew requirements | 0  1 | 0  1 | No measures (i.e., no restrictions)  Required |
| ***Domain: Economic response policies*** | | | | |
| 1 | Income support | 0  1  2  3 | 0  0.33  0.66  1 | No measures (i.e., no restrictions)  Unemployment financial support only  Other income support only  Both unemployment financial support and other income support |
| 2 | Housing support | 0  1 | 0  1 | No measures (i.e., no restrictions)  Support |
| 3 | Utility support | 0  1 | 0  1 | No measures (i.e., no restrictions)  Support |
| 4 | Paid sick leave | 0  1 | 0  1 | No measures (i.e., no restrictions)  Support |
| 5 | Nutrition support | 0  1 | 0  1 | No measures (i.e., no restrictions)  Support |
| ***Domain: Public health policies*** | | | | |
| 1 | Public information campaigns | 0  1  2 | 0  0.5  1 | No COVID-19 public information campaign  Public officials urging caution about COVID-19  Coordinated public information campaign |
| 2 | Testing policy | 0  1  2  3 | 0  0.33  0.66  1 | No testing policy  Only those who both (a) have symptoms AND (b) meet specific criteria  Testing of anyone showing COVID-19 symptoms  Open public testing (e.g., testing available to asymptomatic people) |
| 3 | Contact tracing | 0  1  2 | 0  0.5  1 | No contact tracing  Limited contact tracing (not done for all cases)  Comprehensive contact tracing (done for all identified cases) |
| 4 | Indoor facial coverings | 0  1  2  3 | 0  0  0.5  1 | No policy  Ban on mask mandates (partial or total)  Required in some specified indoor shared spaces outside the home  Required in all indoor shared spaces outside the home |
| 5 | Outdoor facial coverings | 0  1  2  3 | 0  0  0.5  1 | No policy  Ban on mask mandates (partial or total)  Required in some specified outdoor shared spaces outside the home  Required in all outdoor shared spaces outside the home |
| 6 | Vaccination availability by group | N/A | 0-1 | Multiple responses allowed with 28 response options for age groups and high-risk groups (e.g., racial minorities; pregnant people; health care workers).  We first computed the proportion of age groups and the proportion of high-risk groups for whom vaccinations were available. We used the greater of these two values as the rescaled value in score calculation. |
| 7 | Vaccination availability by organization/location | N/A | 0-1 | Multiple responses allowed with 13 response options for clinical locations (e.g., hospitals), pharmacies, public settings (e.g., grocery stores), educational institutions, and mass vaccination sites.  We calculated the proportion of all locations at which vaccinations were available and used this as the rescaled value in score calculation. |
| 8 | Proof of vaccination or negative test for indoor space/event access | 0  1  2  3 | 0  0  0.5  1 | No policy  Ban on policies restricting access  Restricted access to indoor shared spaces based on proof of vaccination or recent negative COVID-19 test in only limited situations  Restricted access to indoor shared spaces based on proof of vaccination or recent negative COVID-19 test in all situations (minor exceptions allowed) |

Source: Wright et al., 2025

| **Supplemental Table 2.** Sample characteristics of BRFSS participants in non-UCCP counties, 2020-2021 | |  |
| --- | --- | --- |
|  | **Mean (SD) or Percent** | |
| N | 526,081 | |
| *Covariates* |  | |
| Age (years) |  | |
| 18-24 | 5.6 | |
| 25-34 | 9.6 | |
| 35-44 | 12.3 | |
| 45-54 | 15.0 | |
| 55-64 | 19.8 | |
| 65+ | 37.6 | |
| Female | 54.2 | |
| Race/ethnicity |  | |
| NH White | 80.9 | |
| Hispanic | 7.1 | |
| NH Black or African American | 5.2 | |
| NH Asian | 1.5 | |
| NH Other^a^ | 5.3 | |
| Education |  | |
| High school or less | 35.1 | |
| Some college or technical school | 28.8 | |
| Graduated college or technical school | 36.1 | |
| Employed | 49.6 | |
| Health insurance coverage | 93.3 | |
| Married or in a couple | 57.5 | |
| *Outcomes*^b^ |  | |
| Frequent mental distress | 12.4 | |
| Any exercise | 74.5 | |
| Current smoker | 14.5 | |
| Any alcohol use | 49.5 | |
| Any binge alcohol use | 12.9 | |
| Any heavy alcohol use | 6.1 | |
| Number of poor physical health days | 3.8 (8.4) | |
| Number of poor mental health days | 3.9 (8.1) | |
| Abbreviations: BRFSS = Behavior Risk Factor Surveillance System; UCCP = US COVID-19 County Policy Database; NH = Non-Hispanic. Table presents percentages for categorical variables and mean (standard deviation) for continuous variables.  ^a^ NH Other includes the following groups collapsed due to small sample size: American Indian or Alaskan Native, Native Hawaiian or other Pacific Islander, Multiracial, and “other race” groups.  ^b^ All outcomes are in reference to the past 30 days, except current smoker which asks about smoking behaviors practiced “now.” | |  |

| **Supplemental Table 3.** Variation in past-8-week policy exposures and continuous health outcomes before (i.e., original) and after accounting for county and month fixed effects (i.e., residual): BRFSS-UCCP data, 2020-2021 (n=287,141). | | | | | |
| --- | --- | --- | --- | --- | --- |
| **Variable** | **Original minimum** | **Original maximum** | **Original standard deviation** | **Residual standard deviation** | **Percent change** |
| Containment policy score | 0.0 | 11.8 | 3.1 | 1.2 | -62.8 |
| Economic policy score | 0.0 | 5.0 | 1.4 | 0.6 | -58.1 |
| Public health policy score | 0.0 | 7.3 | 1.7 | 0.5 | -70.6 |
| Total policy score | 0.0 | 21.0 | 4.8 | 1.5 | -67.9 |
| N days poor physical health | 0.0 | 30.0 | 7.8 | 7.8 | -0.4 |
| N days poor mental health | 0.0 | 30.0 | 8.1 | 8.1 | -0.3 |
| Abbreviations: BRFSS = Behavioral Risk Factor Surveillance System; UCCP = US COVID-19 County Policy Database. Both outcomes are in reference to the past 30 days. | | | | | |

**References**

Beatty, A. L., Peyser, N. D., Butcher, X. E., Carton, T. W., Olgin, J. E., Pletcher, M. J., & Marcus, G. M. (2021). The COVID-19 Citizen Science Study: Protocol for a Longitudinal Digital Health Cohort Study. *JMIR Research Protocols*, *10*(8), e28169. https://doi.org/10.2196/28169

CDC. (2024a, August 29). *BRFSS Survey Data & Documentation*. https://www.cdc.gov/brfss/data_documentation/index.htm

CDC. (2024b, October 22). *Social Vulnerability Index*. Place and Health - Geospatial Research, Analysis, and Services Program (GRASP). https://www.atsdr.cdc.gov/place-health/php/svi/index.html

Hamad, R., Lyman, K. A., Lin, F., Modrow, M. F., Ozluk, P., Azar, K. M. J., Goodin, A., Isasi, C. R., Kitzman, H. E., Knight, S. J., Marcus, G. M., McMahill-Walraven, C. N., Meissner, P., Nair, V., O’Brien, E. C., Olgin, J. E., Peyser, N. D., Sylwestrzak, G., Williams, N., … Carton, T. (2022). The U.S. COVID-19 County Policy Database: A novel resource to support pandemic-related research. *BMC Public Health*, *22*(1), 1882. https://doi.org/10.1186/s12889-022-14132-6

Hamad, R., Pletcher, M. J., & Carton, T. (2024). *United States COVID-19 County Policy Database, 2020-2021* [Dataset]. Inter-university Consortium for Political and Social Research. https://doi.org/10.3886/ICPSR39109.v1

Liu, J., Jiang, N., Fan, A. Z., & Weissman, R. (2018). Alternatives in Assessing Mental Healthcare Disparities Using the Behavioral Risk Factor Surveillance System. *Health Equity*, *2*(1), 199–206. https://doi.org/10.1089/heq.2017.0056

National Institute on Alcohol Abuse and Alcoholism. (2025, February). *Alcohol’s Effects on Health*. NIH. https://www-niaaa-nih-gov.ezp-prod1.hul.harvard.edu/alcohols-effects-health/alcohol-drinking-patterns#:~:text=NIAAA%20defines%20heavy%20drinking%20as,or%20more%20drinks%20per%20week

Wright, E., Dore, E. C., Jackson, K. E., Wang, G., Pletcher, M. J., Carton, T. W., & Hamad, R. (2025). County-Level COVID-19 Policy Comprehensiveness and Adult Behavioral Health during 2021. *Journal of Urban Health*. https://doi.org/10.1007/s11524-025-00982-z
